# The outcome of major psychiatric and substance use disorders as an index of genetic risk and genetic heterogeneity

**DOI:** 10.1017/S0033291725101116

**Published:** 2025-08-12

**Authors:** Kenneth S. Kendler, Henrik Ohlsson, Jan Sundquist, Kristina Sundquist

**Affiliations:** 1 Virginia Institute for Psychiatric and Behavioral Genetics, Virginia Commonwealth University, Richmond, VA, USA; 2 Department of Psychiatry, Virginia Commonwealth University, Richmond, VA, USA; 3 Center for Primary Health Care Research, Department of Clinical Sciences, Lund University, Malmö, Sweden; 4 University Clinic Primary Care Skåne, Region Skåne, Sweden

**Keywords:** anxiety disorders, bipolar disorder, genetic heterogeneity, genetic risk, psychiatric outcomes, schizophrenia, social outcomes, substance use disorders

## Abstract

**Background:**

We investigate whether, in Swedish national registers, social and psychiatric outcomes for six major psychiatric and substance disorders – drug use disorder (DUD), alcohol use disorder (AUD), major depression (MD), bipolar disorder (BD), anxiety disorder (AD), and schizophrenia (SZ) – reflect the primary genetic risk for each disorder and the level of genetic heterogeneity.

**Methods:**

We utilize Genetic Risk Ratios – defined as the ratio of the genetic risk for secondary disorders to the genetic risk for the primary disorder – derived from Family Genetic Risk Scores. Poor social outcome was defined by a common factor of four variables: receipt of social welfare, sick leave, early retirement pension, and residence in a socially deprived area. Psychiatric outcome was defined as days of inpatient psychiatric hospitalization.

**Results:**

With poorer social outcomes, the primary genetic risks rose robustly for all disorders except SZ, as did the secondary genetic risks for DUD, AUD, and attention-deficit hyperactivity disorder. With poorer psychiatric outcomes, available only for BD and SZ, the primary genetic risks increased sharply. Overall, MD, AD, and BD became substantially more genetically heterogenous as their social outcomes became poorer, while for AUD, DUD, and SZ, the increase in heterogeneity was more modest. By contrast, with poorer psychiatric outcome, genetic risks for SZ became substantially more genetically homogeneous, with a similar but less robust trend seen for BD.

**Conclusions:**

Despite important differences between our primary disorders, social and psychiatric outcomes are often robust indices of genetic risk and can reflect the levels of genetic heterogeneity.

## Introduction

Since the time of Kraepelin, outcome has been a central feature of descriptive psychiatry and has played a major role in our diagnostic system (Kraepelin, 1899b; Kraepelin, 1990), with poor outcome being, for example, a defining feature of schizophrenia (SZ) (E. Kraepelin, 1899c). In 1933, Bruno Schulz examined the relationship between the long-term outcome of SZ patients and the risk for SZ in their siblings, finding at most a weak relationship (Kendler & Klee, 2022; Schulz, 1933). Since that time, the outcome has occasionally been explored as a possible index of genetic risk with mixed results. Such studies have nearly always been for one disorder at a time and typically in relatively small patient cohorts (Cuesta et al., 2024; Fusar-Poli et al., 2022; Mistry et al., 2018a, 2018b; Sakamoto et al., 2016). One exception to this trend was a well-powered study by Kämpe et al., who, in a Finnish cohort, showed that high polygenic risk score (PRS) for SZ was associated with a more severe course, as indexed by the total days in a hospital (Kämpe et al., 2024).

In this report, using large Swedish national samples of six *primary* disorders (i.e. major depression [MD], anxiety disorder [AD], alcohol use disorder [AUD], drug use disorder [DUD], bipolar disorder [BD], and SZ), we begin by examining the relationship between genetic risk for a range of what we term ‘*secondary* disorders’ defined here as the six disorders we examine individually plus attention-deficit hyperactivity disorder (ADHD), autism spectrum disorder (ASD), and low educational attainment (LEA) – calculated by the Family Genetic Risk Score (FGRS) (Kendler, Ohlsson, Sundquist, & Sundquist, 2021a, 2021b; 2023a; 2023b) – and poor *social outcome.* Poor social outcome is defined by a common factor derived from four indices, such as receipt of social welfare, frequent sick leave, early retirement pension, and residence in a socially deprived area. In addition, for the two disorders where psychiatric hospitalization was common (SZ and BD), we assessed what we term “psychiatric outcome” by measuring the total days of inpatient care (Kämpe et al., 2024). Next, we calculated “Genetic Risk Ratio” (GRR) as the ratio of the genetic risk for secondary disorders to the genetic risk for the primary disorder. Secondary disorders were selected because of likely genetic relationships with particular primary disorders. In addition, because of its importance for the outcome more generally, we include in all these analyses the genetic risk for LEA.

The GRR statistic is critical to our study, and functions as a quantitative index of genetic heterogeneity. That is, a rising GRR with worse outcome reflects increasing genetic *heterogeneity*, while a falling GRR with worse outcome reflects greater genetic *homogeneity.* This is because a rising GRR means, by definition, that the genetic risk for the secondary disorder is increasing more rapidly with poor outcome than the genetic risk for the primary disorder. The reverse would be true with a GRR that became smaller with worsening outcomes. We will see examples of both these patterns of results in this article.

For each of our six primary disorders, we ask two questions. First, to what extent does the genetic risk for that disorder vary as a function of outcome? Second, are varying levels of outcome an index of genetic heterogeneity, which would be seen in the profile of genetic risk scores for our secondary disorders among affected individuals differing in their level of outcome? For BD and SZ, we addressed a third question: what was the pattern of genetic findings when we examined their psychiatric outcomes, and was it similar to that seen with their social outcomes?

## Methods

**Figure 1.**
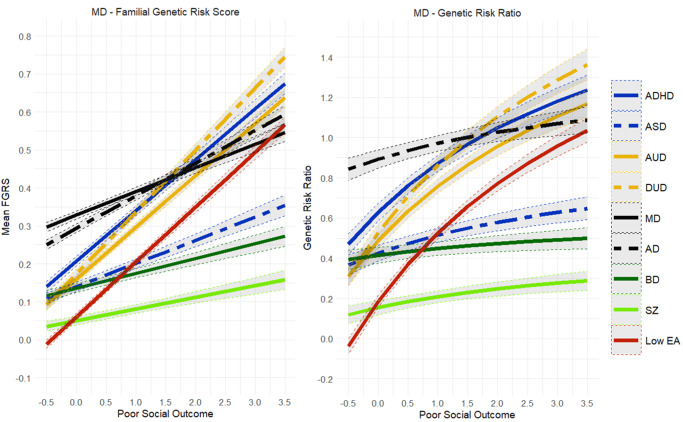
Mean Family Genetic Risk Score (FGRS) and Genetic Risk Ratio (GRR), both ±95% CIs (on the *y*-axis) in individuals with *major depression* as a function of their standardized factor scores for poor social outcome (on the *x*-axis). The FGRS figure includes the primary disorder (here major depression) and the secondary disorders/traits (ADHD, autism spectrum disorder (ASD), alcohol use disorder (AUD), drug use disorder (DUD), anxiety disorder (AD), bipolar disorder (BD), schizophrenia (SZ), and low educational attainment (LEA). The GRR figure, which only includes the secondary disorders/traits, presents the GRRs (see Methods section for definition).

We collected information on individuals from Swedish population-based registers with national coverage linking each person’s unique personal identification number, which, to preserve confidentiality, was replaced with a serial number by Statistics Sweden (for details, see Supplementary Appendix Table 1 and Figure 1). We secured ethical approval for this study from the Regional Ethical Review Board in Lund, and participant consent was not required (No. 2008/409 and later amendments). From the Swedish registers, we identified all individuals born in Sweden between 1972 and 1980 to Swedish-born parents. Inclusion was restricted to individuals with available data on four key indicators of poor social outcomes: early retirement, social welfare receipt, sick leave benefits, and residence in a deprived area (for details, see Supplementary Appendix Table 2). For each individual, we calculated the number of years between the ages of 34 and 38 years during which they were registered for any of these indicators. Years of registration were then categorized into three groups: 0 years, 1 year, or more than 2 years. Early retirement was categorized into two groups: 0 years or 1 or more years. Using these indicators, we performed a factor analysis to create a composite measure of poor social outcome (for details, see Supplementary Appendix Tables 3a and 3b). Standardized factor scores were calculated for each individual and used as an indicator of poor social outcome.

**Figure 2.**
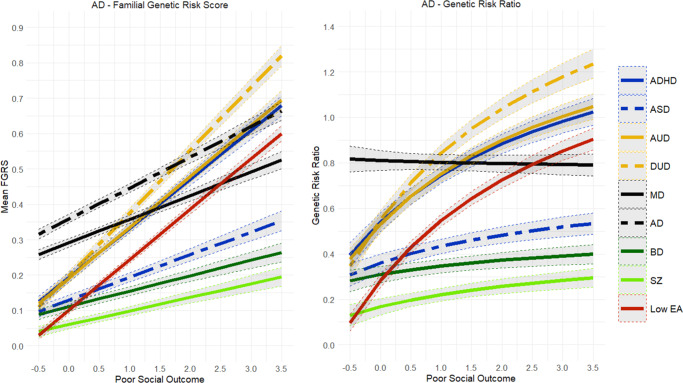
Mean Family Genetic Risk Score (FGRS) and Genetic Risk Ratio (GRR), both ±95% CIs (on the *y*-axis) in individuals with *anxiety disorders* as a function of their standardized factor scores for poor social outcome (on the *x*-axis). The FGRS figure includes the primary disorder (here anxiety disorders) and the secondary disorders/traits (ADHD, autism spectrum disorder (ASD), alcohol use disorder (AUD), drug use disorder (DUD), major depression (MD), bipolar disorder (BD), schizophrenia (SZ), and low educational attainment (LEA). The GRR figure, which only includes the secondary disorders/traits present, the GRRs (see Methods section for definition).

Missingness for these measures was modest. Of all individuals born in Sweden between 1972 and 1980 to Swedish-born parents (*N* = 700,626), information on poor social outcomes was available for 95.2% of the cohort. Among the 33,684 individuals for whom data were missing, 13,901 had died before the age of 38 years, and 19,677 had emigrated before turning 38. In total, data were missing for only 106 individuals aged 34–38 years who were still residing in Sweden.

Next, we constructed six separate datasets for individuals diagnosed with one of our six primary disorders: MD, AD, BD, SZ, AUD, and DUD. Information on these diagnoses was derived from national patient and primary care registers using diagnostic codes (details provided in Supplementary Appendix Table 2). The registration had to occur before the age of 34 years to ensure the poor social outcome variables were recorded after registration for the primary disorder. In these datasets, we also included individual FGRS for each of the primary disorders and two secondary disorders – ADHD and ASD – as well as for LEA.

FGRS were calculated based on morbidity risks for disorders observed in first- through fifth-degree relatives, accounting for cohabitation effects. These scores reflect phenotypes within extended pedigrees rather than molecular genetic data. Further details on the data sources and calculation methods are available in Supplementary Appendix Table 4. We regressed the FGRS for each of the six primary disorders (MD, AD, BD, SZ, AUD, and DUD), as well as the two secondary disorders (ADHD and ASD) and LEA on the standardized factor scores for poor social outcomes accounting for year of first registration for the primary disorder. This approach allowed us to assess the association between genetic liability, as captured by FGRS, on their social outcome. In addition, we used these models to estimate the predicted FGRS values across different levels of the composite measure of poor social outcome. Thereafter, we calculated the GRRs, where the denominator was the FGRS for the primary disorder, and the numerator was the FGRS for the secondary disorders. Thus, the GRRs reflected the ratio of genetic risks for the secondary and primary disorder across levels of social outcome for the primary disorder. We also present the linear slope of all FGRS across the composite measure.

In the second part of our analysis, which focused on psychiatric outcomes, we constructed two separate datasets: one for individuals diagnosed with BD and another for individuals diagnosed with SZ. Here, we included all individuals registered with these diagnoses who were born in Sweden to Swedish-born parents. In these analyses, we did not restrict our samples to specific age ranges. Follow-up time ended on December 31, 2018. For SZ, the mean year of birth (YoB) was 1951 (SD: 19.3), the mean age at registration was 42.0 (SD: 16.5), and the mean follow-up time was 25.6 years (SD: 14.6). The corresponding numbers for BD were as follows: the mean YoB was 1960 (SD: 23.3), the mean age at registration was 42.9 (SD: 17.5), and the mean follow-up time was 15.1 years (SD: 13.2).

For each individual, controlling for the year of registration, we calculated the total number of days spent in psychiatric hospitalization by summing the duration (difference between admission and discharge dates) of all hospital stays linked to BD or SZ diagnoses. By correcting for the year of first registration in these analyses, we thereby control, at least partially, for the general decline in length of psychiatric hospitalizations over our follow-up period. Registrations for nonhospital care were assigned a value of 0. To analyze this data, we standardized the total hospitalization days into a *z*-score with a mean of 0 and a standard deviation of 1. We then applied the same modeling approach used for poor social outcomes, replacing the composite measure of poor social outcomes with the standardized hospitalization variable, our measure of psychiatric outcome. All statistical analyses were performed using SAS 9.4 (SAS Institute, 2012) and/or R 4.3.1 (R Core Team, 2023).

## Results

### Social outcome

Table 1 depicts the sample sizes available for our six primary diagnoses, which ranged from 1,453 for SZ to 46,570 for MD. Consistent with expectations, the median standardized social outcome scores across diagnostic categories were lowest for MD, AD, and AUD, intermediate for BD and DUD, and highest for SZ.

**Figure 3.**
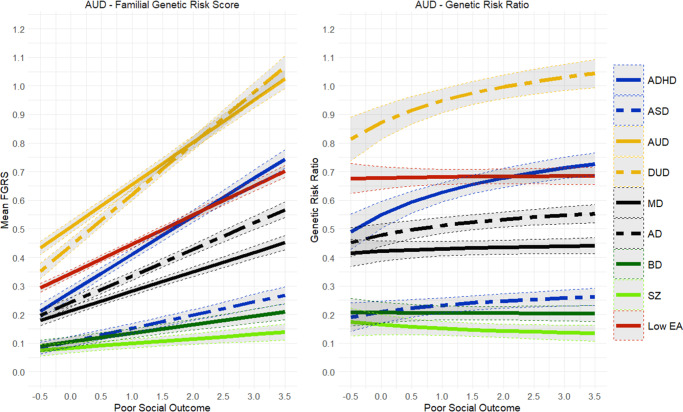
Mean Family Genetic Risk Score (FGRS) and Genetic Risk Ratio (GRR), both ±95% CIs (on the *y*-axis) in individuals with *alcohol use disorder* as a function of their standardized factor scores for poor social outcome (on the *x*-axis). The FGRS figure includes the primary disorder (here alcohol use disorder) and the secondary disorders/traits (ADHD, autism spectrum disorder (ASD), anxiety disorders (AD), drug use disorder (DUD), major depression (MD), bipolar disorder (BD), schizophrenia (SZ), and low educational attainment (LEA). The GRR figure, which only includes the secondary disorders/traits, presents the GRRs (see Methods section for definition).

**Table 1.** Sample sizes for our main disorders and their median score for the social outcome factor

Disorder	*N* for social outcome	Factor score: Median (25–75)	*N* for psychiatric outcome
MD	46,570	−0.13 (−0.27; 0.40)	–
AD	39,956	−0.13 (−0.27; 0.40)	–
AUD	20,916	−0.13 (−0.27; 1.32)	–
DUD	17,957	0.26 (−0.27; 3.33)	–
BD	3,526	0.26 (−0.20; 1.25)	89,571
SZ	1,453	0.72 (0.72; 1.25)	34,135

Results for MD are seen in Figure 1 where, as in all figures, the results for the FGRS are presented in the left half of the figure and GRR in the right half. The *x*-axis here is our measure of the social outcome of the MD cases. For MD, all nine FGRS values increased as the outcome worsened, with the steepest slopes seen for FGRS_DUD_, FGRS_ADHD_, FGRS_AUD,_ and FGRS_LEA_. By comparison, the slopes for FGRS_MD_ and FGRS_AD_ were shallower. For the GRR analyses, the genetic heterogeneity of MD increased considerably as the outcome worsened, with higher genetic risks for ADHD, AUD, and DUD and more modestly increased risks for AD and SZ. That is, as the outcome worsened for MD, the genetic risk for all these disorders increased more rapidly than did the genetic risk for MD. Poor outcome in MD was also associated with a higher genetic risk for LEA. Only the GRR for FGRS_BD_ changed little across levels of social outcome.

**Figure 4.**
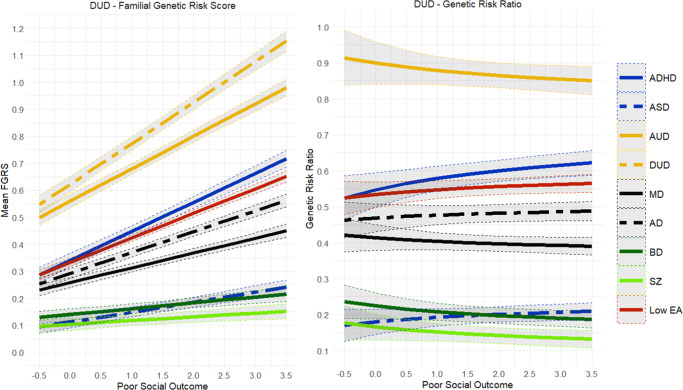
Mean Family Genetic Risk Score (FGRS) and Genetic Risk Ratio (GRR), both ±95% CIs (on the *y*-axis) in individuals with *drug use disorder* as a function of their standardized factor scores for poor social outcome (on the *x*-axis). The FGRS figure includes the primary disorder (here drug use disorder) and the secondary disorders/traits (ADHD, autism spectrum disorder (ASD), anxiety disorders (AD), alcohol use disorder (AUD), major depression (MD), bipolar disorder (BD), schizophrenia (SZ), and low educational attainment (LEA). The GRR figure, which only includes the secondary disorders/traits, presents the GRRs (see Methods section for definition).

The findings for AD (Figure 2) closely resembled those seen for MD and only differed in that the GRR for FGRS_MD_ slowly declined with increasingly poor outcomes. By contrast, the results for AUD and DUD (Figures 3 and 4) were quite different. The genetic risks for AUD and DUD increased more rapidly with worsening outcome than any of the secondary disorders, with the single exception of FGRS_ADHD_. With increasingly poor outcomes, the genetic profile for cases of AUD showed substantial increases in FGRS_DUD_ and FGRS_ADHD_, but little change otherwise, including the genetic risk for LEA. A very similar pattern is seen for DUD in Figure 4.

**Figure 5.**
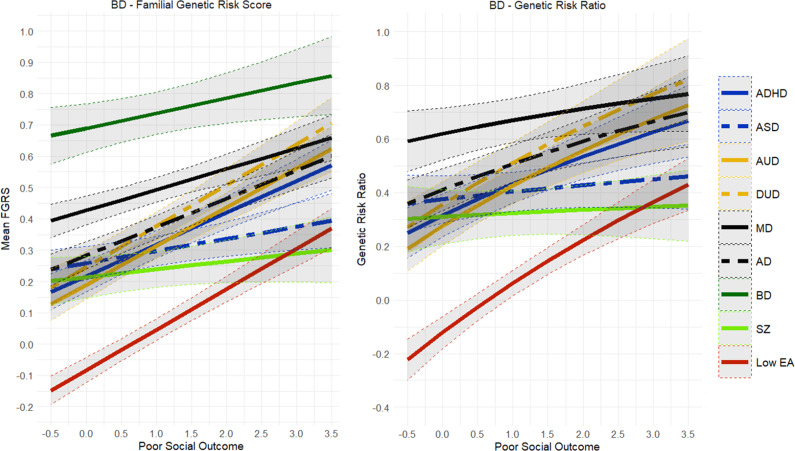
Mean Family Genetic Risk Score (FGRS) and Genetic Risk Ratio (GRR), both ±95% CIs (on the *y*-axis) in individuals with *bipolar disorder* as a function of their standardized factor scores for poor social outcome (on the *x*-axis). The FGRS figure includes the primary disorder (here bipolar disorder) and the secondary disorders/traits (ADHD, autism spectrum disorder (ASD), anxiety disorders (AD), alcohol use disorder (AUD), drug use disorder (DUD), major depression (MD), schizophrenia (SZ), and low educational attainment (LEA). The GRR figure, which only includes the secondary disorders/traits, presents the GRRs (see Methods section for definition).

**Figure 6.**
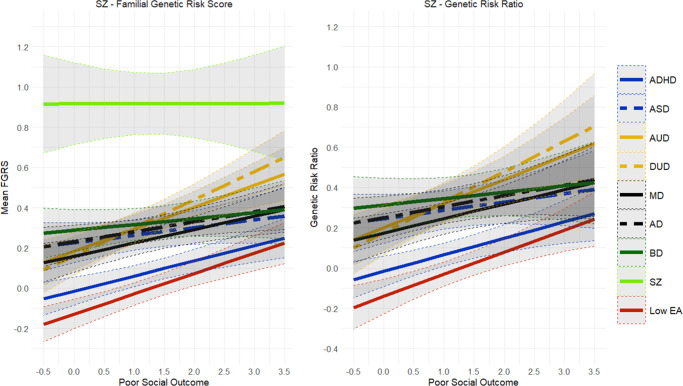
Mean Family Genetic Risk Score (FGRS) and Genetic Risk Ratio (GRR), both ±95% CIs (on the *y*-axis) in individuals with *schizophrenia* as a function of their standardized factor scores for poor social outcome (on the *x*-axis). The FGRS figure includes the primary disorder (here schizophrenia) and the secondary disorders/traits (ADHD, autism spectrum disorder (ASD), anxiety disorders (AD), alcohol use disorder (AUD), drug use disorder (DUD), major depression (MD), bipolar disorder (BD), and low educational attainment (LEA). The GRR figure, which only includes the secondary disorders/traits, presents the GRRs (see Methods section for definition).

Results for the social outcome for BD (Figure 5) differed from those seen with MD, AD, AUD, and DUD in two important ways (in addition to much larger confidence intervals (CIs) because of the smaller sample size). First, the rise in FGRS_BD_ with increasingly poor outcomes was much more modest than that seen with prior disorders. Second, the genetic risk for LEA was negative for better outcome BD cases and increased sharply with poorer outcomes. Furthermore, GRRs increased appreciably for MD, AD, DUD, AUD, ADHD, and LEA, with more modest increases observed for SZ and ASD, a pattern quite different from that seen for AUD and DUD.

Finally, findings for FGRS_SZ_ (Figure 6) were also unique in showing virtually no change in the primary genetic risk with increasingly poor outcomes. Mild increases were seen in FGRS_DUD_ and FGRS_AUD_. FGRS_ADHD_ shifted from negative in good social outcome cases to low and positive in SZ with poor social outcomes. The GRR analyses found modest increases in the GRR with increasingly poor social outcomes, particularly for AUD, DUD, and MD.

**Figure 7.**
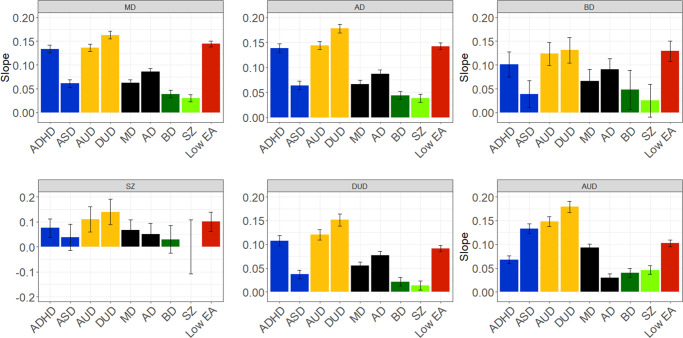
The slope (±95% CIs) for the Family Genetic Risk Score (FGRS) analyses examining social outcome of major depression (MD), anxiety disorders (AD), bipolar disorder (BD), schizophrenia (SZ), drug use disorder (DUD), and alcohol use disorder (AUD). LEA stands for genetic risk for low educational attainment, and ASD is the genetic risk for autism spectrum disorder.

Figure 7 gives linear slope estimates and 95% CIs for all the findings presented in Figures 1–6. Most findings are statistically significant, except for some in the rarest of our disorders, such as SZ.

### Psychiatric outcomes

**Figure 8.**
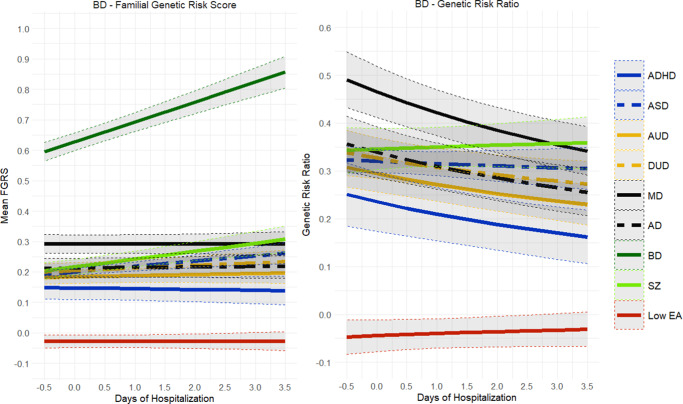
Mean Family Genetic Risk Score (FGRS) and Genetic Risk Ratio (GRR), both ±95% CIs (on the *y*-axis) in individuals with bipolar disorder as a function of their psychiatric outcome, operationalized by the total days of inpatient hospitalization (on the *x*-axis). The FGRS figure includes the primary disorder (here Bipolar Disorder), and the secondary disorders/traits (ADHD, autism spectrum disorder (ASD), anxiety disorders (AD), alcohol use disorder (AUD), drug use disorder (DUD), major depression (MD), schizophrenia (SZ), and low educational attainment (LEA). The GRR figure, which only includes the secondary disorders/traits, presents the GRRs (see Methods section for definition).

Because we could sample a much broader birth cohort for these analyses, our sample sizes for BD (*n* = 89,571) and SZ (*n* = 34,135) were substantially larger than those available for our social outcome analyses (Table 1). Of note, 51.9% of our BD and 81.5% of our SZ cohort had been psychiatrically hospitalized. The results for our psychiatric outcome analyses for BD are seen in Figure 8 and showed substantial differences from the social outcome analyses depicted in Figure 5. Nearly all the genetic risks in BD patients were stable across worsening psychiatric outcomes, with two exceptions. FGRS_BD_ increased moderately, and FGRS_SZ_ increased modestly. GRR values fell for nearly all disorders, with increasingly poor psychiatric outcomes, with only one exception – a modestly rising GRR for FGRS_SZ_.

**Figure 9.**
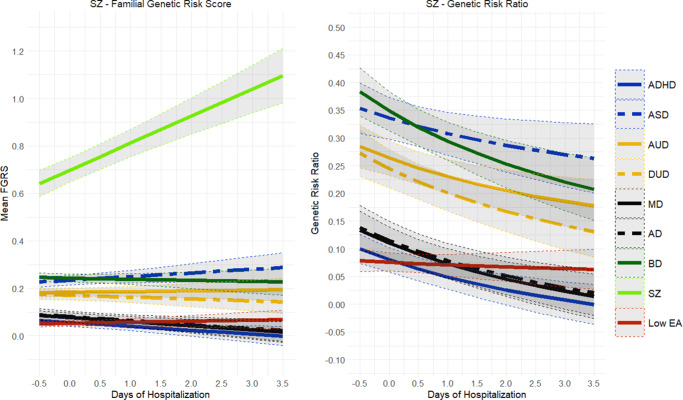
Mean Family Genetic Risk Score (FGRS) and Genetic Risk Ratio (GRR), both ±95% CIs (on the *y*-axis) in individuals with schizophrenia as a function of their psychiatric outcome, operationalized by the total days of inpatient hospitalization (on the *x*-axis). The FGRS figure includes the primary disorder (here schizophrenia) and the secondary disorders/traits (ADHD, autism spectrum disorder (ASD), anxiety disorders (AD), alcohol use disorder (AUD), drug use disorder (DUD), major depression (MD), bipolar disorder (BD), and low educational attainment (LEA). The GRR figure, which only includes the secondary disorders/traits, presents the GRRs (see Methods section for definition).

**Figure 10.**
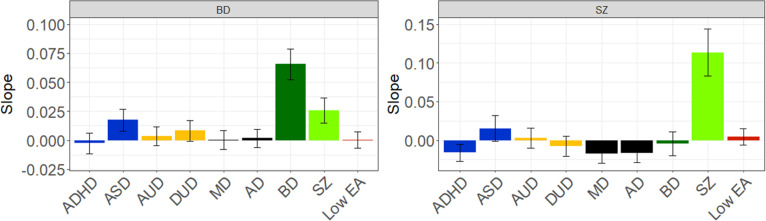
The slope (±95% CIs) for the Family Genetic Risk Score (FGRS) analyses examining psychiatric outcome for bipolar disorder (BD) and schizophrenia (SZ). Other initials used in the figure are: major depression (MD), anxiety disorders (AD), drug use disorder (DUD), alcohol use disorder (AUD), autism spectrum disorder (ASD), and genetic risk for low educational attainment (LEA).

The pattern of results for psychiatric outcome in SZ, seen in Figure 9, also differed dramatically from those seen for social outcomes in Figure 9. The only genetic risk that increased in SZ patients with poorer psychiatric outcomes was FGRS_SZ_. Noteworthy in the GRR analyses was the declining levels of GRR for nearly all psychiatric disorders, with worsening psychiatric outcome for SZ, with the particularly rapid fall in the GRR for BD. Figure 10 gives linear slope estimates and 95% CIs for all the findings presented in Figures 8–9.

## Discussion

The goal of this study was to determine whether dimensions of outcome for a core group of six major psychiatric and substance use disorders impacted the genetic risk for the primary disorder and the genetic heterogeneity of that disorder. This heterogeneity was indexed by the pattern of genetic risks in affected individuals for a range of other psychiatric and substance use disorders, which we here called ‘secondary disorders’. We would like to emphasize, in this empirically rich article, three main patterns of results for our psychiatric disorders that we will review in turn.

First, we saw substantial similarities in the results for variation in social outcomes for MD and AD. In each of these disorders, the genetic risk for the primary disorder nearly doubled in those with the worst social outcome compared to the best. However, the genetic risk for the three externalizing disorders – AUD, DUD, and ADHD – all increased more quickly with poorer social outcomes. As a result, poor social outcomes in MD and AD were more genetically heterogeneous than were those with better outcomes. Of note, while the GRR for FGRS_AD_ rose with poorer outcomes in MD, the GRR for FGRS_MD_ modestly declined with increasingly poor AD outcomes. That is, high genetic risk for AD in MD patients was associated with a poor social outcome, while high MD genetic risk in cases of AD was associated with a relatively good social outcome. This may arise because the genetic liability to MD is for an episodic disorder with relatively good outcome, while the liability to AD is for a more chronic and sometimes impairing condition.

Second, the pattern of results was similar for DUD and AUD. In both disorders, FGRS_AUD_ and FGRS_DUD_ rose rapidly with increasingly poor social outcomes, accompanied by rising genetic risks for ADHD, and more modest increases in FGRS_AD_ and FGRS_MD_. The GRR analyses showed only a slight increase in genetic heterogeneity with poorer social outcomes.

Third, for BD and SZ, because a majority of our subjects with these disorders were psychiatrically hospitalized, we were able to examine both social and psychiatric outcomes – the latter measured by total days spent in a hospital. For both disorders, the results were very different, which supports an observation made many years ago by Strauss and Carpenter about the outcome in SZ:

The different areas of outcome dysfunction: work, symptoms, social relations, and duration of non-hospitalization, seem to operate as open systems, all partly interrelated and affected by psychiatric disorder but each area also affected by variables more specific to it alone. (Strauss & Carpenter, 1972) (p. 739)

Poor social outcome in BD was weakly predicted by FGRS_BD_, but more strongly predicted by genetic risk for AUD, DUD, ADHD, and MD. However, it was not predicted by genetic risk for SZ. Surprisingly, poor social outcome was not predicted in SZ cases by FGRS_SZ_, but, like for BD, it was predicted by greater genetic risks for AUD, DUD, ADHD, and MD. So, for both major disorders, poorer social outcomes were associated with greater genetic heterogeneity, most prominently for externalizing disorders.

Our findings are congruent with increasing evidence that substantial substance use or substance use disorders negatively impact the clinical course of SZ and BP (Faber et al., 2012; Groening et al., 2023; Ouellet-Plamondon, Abdel-Baki, Salvat, & Potvin, 2017; Preuss, Hesselbrock, & Hesselbrock, 2023; Zammit et al., 2008). That is, one likely mediational path for the impact of high genetic risk for AUD and DUD on social outcomes of BD and SZ is via increased risk for the abuse of alcohol and illicit substances.

By contrast, the pattern for results for poor psychiatric outcomes for BD and SZ looked quite different. For both disorders, genetic risk was strongly associated with more days of psychiatric hospitalization, and this was nearly unrelated to other genetic risks. One interesting exception to this pattern was that for BD, increasing FGRS_SZ_ was associated with poorer psychiatric outcome. However, for SZ, lower rates of FGRS_BD_ were associated with poorer outcomes. For SZ, we found robust evidence that poor psychiatric outcome was associated with less genetic heterogeneity. A similar but somewhat weaker pattern was also seen for BD.

These results suggest, in accord with Strauss and Carpenter’s position, that the genetic contribution to poor outcomes in SZ and BD differs substantially across outcome dimensions. For example, while high genetic risk for the externalizing disorders of AUD, DUD, and ADHD contributed strongly to poor social outcomes in SZ, they were nearly unrelated to our psychiatric outcome measure.

How congruent were our findings with the modest prior literature on the association of genetic risk to outcome across our primary disorders? We previously examined the impact of FGRS for MD, AD, AUD, DUD, BD, and nonaffective psychosis on four dimensions of poor social outcome in a differently defined Swedish cohort (Kendler, Ohlsson, Sundquist, & Sundquist, 2024a). About 75% of these associations were significant. No cross-disorder genetic risk scores were examined. Given the overlap in samples and measures, this cannot be regarded as an independent replication of our findings.

Most directly, our results on the psychiatric outcome in SZ directly replicate findings from a large cohort of SZ patients in Finland, where high PRS scores for SZ predicted an ‘increased psychiatric hospitalization burden’ (Kämpe et al., 2024). The best recent review of this small literature, by Fusar-Poli et al. (2022), reported mixed success in PRS scores predicting outcome and/or treatment response in SZ, BD, and MD. They cite one report, broadly congruent with our findings, that a PRS score for ADHD predicted treatment resistance in cases of MD (Fabbri et al., 2021). Sakamoto et al., in a modestly sized Japanese SZ cohort, found individual variants and a SZ PRS to predict both clinical and social outcomes in SZ (Sakamoto et al., 2016). We found no literature relating genetic risk to outcome for AUD and DUD.

We included in our results the genetic risk for LEA because of its likely involvement in the prediction of poor outcomes. This expectation was robustly fulfilled in our study of social outcomes with two interesting nuances. First, FGRS_LEA_ was substantially associated with poor social outcomes in all of our disorders, with the results being particularly strong for MD, AD, AUD, and DUD. This is likely a result of the strong association between LEA and low social class (Broer et al., 2019), which is, in turn, predictive of our poorer social outcome measures, especially receipt of social welfare and living in areas of high social deprivation. Put another way, our findings suggest that a strong predisposition to LEA reduces an individual’s ability to cope well with the adversities of psychiatric illness, rendering them more vulnerable to the associated decline in social functioning. Second, by contrast to the results with social outcome, the level of FGRS_LEA_ was, for both SZ and BD, unrelated to variation in psychiatric outcome.

Finally, what did we learn about the pattern of association of genetic risks for a range of disorders and the outcome of our key psychiatric disorders? Our GRR analyses show that MD, which is a disorder that typically has a relatively good outcome, was more genetically homogeneous in cases with good versus poor social outcomes. Furthermore, in AD cases, high genetic risk for MD was associated with good social outcomes. Our results would suggest that, in part, poor outcomes in MD are more likely to arise in individuals not only with an elevated genetic risk for MD, but with high genetic risks for DUD, AUD, and/or ADHD. The picture for DUD is different, with little change in the GRRs across levels of social outcome, suggesting that for DUD, poor outcome might be driven more by higher FGRS_DUD_ rather than other genetic risks. In terms of social outcome, BD had increased genetic heterogeneity in the poor outcome cases, but the results were quite different for psychiatric outcome, where the poor outcome cases had lower levels of heterogeneity. It is noteworthy that in BD cases, high GRR for MD was associated with poorer social outcome but better psychiatric outcome. Our results for SZ were of particular clinical interest. Our psychiatric outcome results supported Kraepelin’s view that poor outcome was a core component of the SZ syndrome (Kraepelin, 1899a; 1899c; 1990) as the FGRS_SZ_ increased substantially with poor psychiatric outcome cases, and the GRR analyses support a greater genetic homogeneity with worse outcomes. Furthermore, high genetic risk for SZ was associated with poorer psychiatric outcome in BD, while high genetic risk for BD was associated with better psychiatric outcome in SZ. These results suggest that the major genetic contribution to poor psychiatric outcome in SZ is a high level of FGRS_SZ_. The pattern for social outcome in SZ cases is, by contrast, quite different, with the level of FGRS_SZ_ being unrelated to social outcome and genetic heterogeneity increasing with poorer outcome. Thus, the genetic factors contributing to social and psychiatric outcomes in SZ appear to differ substantially.

### Limitations

These results should be interpreted in the context of three potential methodological limitations. First, the validity of our findings is dependent upon the quality of the diagnoses in the Swedish registries. The validity of Swedish hospital diagnoses for SZ and BD is well supported (Ekholm et al., 2005; Lichtenstein et al., 2006; Sellgren et al., 2011) as is the validity of MD based on its prevalence, sex ratio, sibling and twin correlations, and associations with known psychosocial risk factors (Kendler et al., 2018; Sundquist, Ohlsson, Sundquist, & Kendler, 2017). The validities of AUD and DUD are supported by the high rates of concordance across ascertainment methods (Kendler et al., 2018; 2015) and the patterns of resemblance in relatives are similar to those found in personally interviewed samples (Prescott & Kendler, 1999; Tsuang et al., 1996). The diagnosis of ADHD in Sweden is validated by its close relationship with the receipt of stimulant medication (Sundquist, Ohlsson, Sundquist, & Kendler, 2015).

Second, the power of the FGRS as a measure of genetic risk is based on the availability, for Swedish-born individuals residing in Sweden, of extensive high-quality phenotypic data on large numbers of close and extended relatives. Therefore, this statistic is best applied to populations with registry information similar to that of Sweden. Of note, the FGRS differs qualitatively from PRS in that it obtains genetic risk indirectly from rates of illness in relatives and not from DNA variation. These two measures of genetic risk, therefore, derive from independent sources and are potentially complementary. We have formally compared, both empirically and via simulations, the FGRS and PRS (Morten Dybdahl Krebs et al., 2023; Dybdahl Krebs et al., 2024) and shown that both scores behave (i.e. predict outcomes and intercorrelate) as measures of additive genetic liability consistent with expected levels of measurement error.

Third, a substantial proportion of individuals in our sample had multiple primary diagnoses (e.g. both AUD and MD, or both MD and AD). Therefore, we conducted sensitivity analyses that exclude individuals with three relevant pairs of comorbid disorders (i.e. MD and AD, AUD and DUD, and BD and SZ) for social outcomes and only the last pair for psychiatric outcomes. We present these results in Supplementary Appendix Figures 2–8. As can be seen, the changes are generally small, with the largest, but still modest differences seen between cases with AUD with and without comorbid cases of DUD.

## Conclusions

We have found that two distinct dimensions of outcome – social and psychiatric – for a representative set of major psychiatric and substance use disorders are useful indices of both genetic risk and level of genetic heterogeneity. We also observed important differences between the patterns of results for our primary disorders. In nearly all current psychiatric genetic studies, the focus is on a dichotomous diagnostic status: unaffected or affected. Our results suggest that this approach is inefficient, as genetic risk varies as a function of outcome. Our findings are thus consistent with a prior study in Sweden samples showing that the number of episodes of illness (Kendler, Ohlsson, Sundquist, & Sundquist, 2024b) also provides relevant information about the level of genetic risk and genetic heterogeneity of major psychiatric and substance use disorders. Additional meaningful genetic information can be obtained from examining outcomes of the disorders we study, and this information is likely to differ as a function of the dimension of outcome examined.

## Supporting information

Kendler et al. supplementary material Kendler et al. supplementary material

## Data Availability

Kristina Sundquist, MD, PhD, had full access to all the data in the study and takes responsibility for the integrity of the data and the accuracy of the data analysis.

## Supplementary material

The supplementary material for this article can be found at http://doi.org/10.1017/S0033291725101116.
