# Supplementary material for: The outcome of major psychiatric and substance use disorders as an index of genetic risk and genetic heterogeneity
Source: Psychol Med. 2025 Aug 12;55:e233. doi: 10.1017/S0033291725101116 (PMC12360690; doi:10.1017/S0033291725101116)

Appendix FGRS Outcome Ms

**Table 1 Description of Registers**

The **Multi-Generation Register** is a comprehensive dataset comprising individuals who have been registered in Sweden since 1961 or who were born in 1932 or later. These individuals are referred to as index persons, and the register includes links between them and their biological parents. Currently, the register contains information on approximately 11 million index persons. The Multi-Generation Register forms part of the broader Total Population Register, with data sourced from the National Tax Board. Each year, a new version of the register is produced, incorporating new index persons born or immigrating during the year. The data from the Multi-Generation Register can be accessed for research and statistical purposes, offering valuable insights into familial and generational connections within Sweden. Further details about the content and data quality are provided in Statistics Sweden, Background Facts, Population and Welfare Statistics 2017:2, Multi-Generation Register 2016.

The **National Patient Register** (NPR) was established in the 1960s by the National Board of Health and Welfare to collect data on in-patients at public hospitals in Sweden. Initially, the register focused on psychiatric care patients and about 16% of those receiving somatic (general) care, covering only six of Sweden’s 26 county councils. In 1984, the Ministry of Health and Welfare, in collaboration with the Federation of County Councils, decided to make participation in the NPR mandatory for all county councils. By 1987, the register expanded to include all inpatient care across Sweden. Since 2001, NPR has been further broadened to cover outpatient doctor visits, including day surgery and psychiatric care, from both public and private healthcare providers. For more detailed information, you can visit the official site of the National Board of Health and Welfare: https://www.socialstyrelsen.se/en/statistics-and-data/registers/register-information/the-national-patient-register/

We also utilized data from **Primary Care registers**, which is a research dataset containing individual-level information on clinical diagnoses from primary healthcare centers in Sweden. By the end of the follow-up period, this dataset covered almost 100% of the population. Figure 1 below illustrates the percentage of the entire Swedish population residing in counties with access to primary care data over time. For more detailed information on this dataset, refer to the study: Sundquist, J., Ohlsson, H., Sundquist, K., Kendler, K.S. (2017), titled Common adult psychiatric disorders in Swedish primary care where most mental health patients are treated, published in BMC Psychiatry (17:235). The full article can be accessed here: <https://doi.org/10.1186/s12888-017-1381-4>.

The Swedish Longitudinal Integration Database for health insurance and labor market studies (LISA) - LISA covers the adult Swedish population aged ≥ 16 years registered on December 31 each year since 1990 (since 2010 individuals aged ≥ 15 years). The database was launched in response to rising levels of sick leave in the country. Participation in Swedish government-administered registers such as LISA is compulsory, and hence selection bias is minimized. For more information see https://link.springer.com/article/10.1007/s10654-019-00511-8 and https://www.scb.se/LISA

**Figure 1 Percentage of the entire Swedish population that resides in counties with primary care data by Year**

*
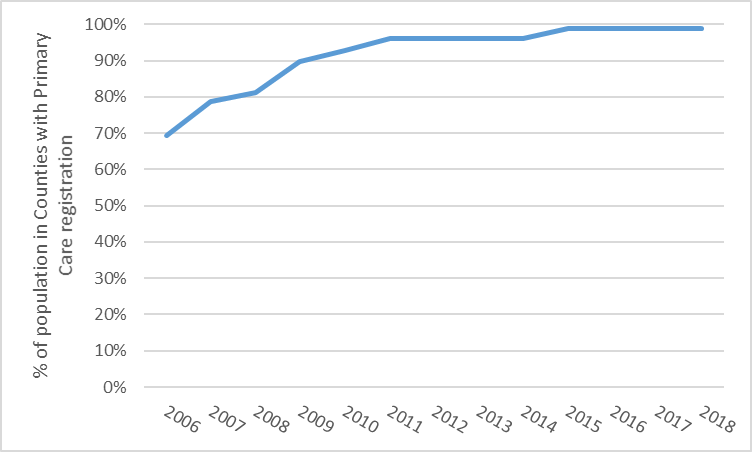
*

**Table 2 Definition of Variables**

|  | Registers Used | Definition |
| --- | --- | --- |
| Schizophrenia (SZ) | The Swedish Hospital Discharge Register (coverage 1997-2018); Outpatient Care Register (national coverage 2001-2018); Primary Care Registry (Partly coverage from 1999-2018) | ICD-8: 295.1, 295.2, 2953, 295.9, 295.6; ICD-9: 295B, 295C, 295D, 295G, 295X; ICD-10: F200, F201, F202, F203, F205, F209 |
| Anxiety Disorder (AD) | The Swedish Hospital Discharge Register (coverage 1973-2018); Outpatient Care Register (national coverage 2001-2018); Primary Care Registry (Partly coverage from 1999-2018) | ICD-8: 300.0, 300.2; ICD-9: 300A, 300C; ICD-10: F40, F41 |
| Major Depression (MD) | The Swedish Hospital Discharge Register (coverage 1973-2018); Outpatient Care Register (national coverage 2001-2018); Primary Care Registry (Partly coverage from 1999-2018) | ICD-8: 296.2, 298.0, 300.4; ICD-9: 296.2, 296.4, 298.0, 300.4; ICD-10: F32, F33. |
| Bipolar Disorder (BD) | The Swedish Hospital Discharge Register (coverage 1973-2018); Outpatient Care Register (national coverage 2001-2018); Primary Care Registry (Partly coverage from 1999-2018) | ICD-8: 296.1, 296.3, 296.8, 296.9, 298.1; ICD-9: 296A, 296C, 296D, 296E, 296W, 298B; ICD-10: F30, F31 |
| Drug Use Disorder (DUD) | The Swedish Hospital Discharge Register (coverage 1973-2018); Outpatient Care Register (national coverage 2001-2018); Primary Care Registry (Partly coverage from 1999-2018); the Swedish Criminal Register (1973-2018) and the Swedish Suspicion Register (1998-2018) | DUD was identified in the Swedish medical and mortality registries by ICD codes (ICD8: Drug dependence (304); ICD9: Drug psychoses (292) and Drug dependence (304); ICD10: Mental and behavioral disorders due to psychoactive substance use (F10-F19), except those due to alcohol (F10) or tobacco (F17)); in the Suspicion Register by codes 3070, 5010, 5011, and 5012, that reflect crimes related to DA; and in the Crime Register by references to laws covering narcotics (law 1968:64, paragraph 1, point 6) and drug-related driving offences (law 1951:649, paragraph 4, subsection 2 and paragraph 4A, subsection 2). |
| Alcohol Use Disorder (AUD) | The Swedish Hospital Discharge Register (coverage 1973-2018); Outpatient Care Register (national coverage 2001-2018); Primary Care Registry (Partly coverage from 1999-2018), and the Swedish Criminal Register (1973-2018) and the Swedish Suspicion Register (1998-2018) | Alcohol Use Disorder (AUD) was identified in the Swedish medical and mortality registries by ICD codes: ICD9: V79B, 305A, 357F, 571A-D, 425F, 535D, 291, 303, 980; ICD 10: E244, G312, G621, G721, I426, K292, K70, K852, K860, O354, T51, F10); in the Crime Register by codes 3005, 3201, which reflect crimes related to alcohol abuse; in the Suspicion Register by codes 0004, 0005 (Only those individuals with at least two alcohol-related crimes or suspicion of crimes from both Crime Register and Suspicion Register were included). |
| ADHD | The Swedish Hospital Discharge Register (coverage 1973-2017); Outpatient Care Register (national coverage 2001-2017); Primary Care Registry (Partly coverage from 1999-2017) | ICD-9: 314; ICD-10: F90 |
| Autism spectrum disorder (ASD) | The Swedish Hospital Discharge Register (coverage 1973-2017); Outpatient Care Register (national coverage 2001-2017); Primary Care Registry (Partly coverage from 1999-2017) | ICD-9: 299; ICD-10: F840, F841, F845, F849 |
| Low Education attainment | The National School Registry, Multigenerational Register, LISA database | For all individuals in our sample we used the Multigenerational Register to identify all 1^st^ to 5^th^ degree relatives. For these relatives, we used information on Average grade point at age 18/19, Average grade point at age 16 and Number of years of education.  Number of years of education are measured in 7 different levels  1 Pre-high school < 9 years  2 High School 9 years  3 Upper Secondary School < 3 years  4 Upper Secondary School 3 years  5 Post-secondary education < 3years  6 Post-secondary education 3 years or more  7 Research education (PhD).  All three educational variables are standardized with mean 0 and SD 1. For all relatives we took the mean Z-score for future calculations. For parents and siblings we corrected for cohabitation effects. To estimate the cohabitation effect (i.e. “shared environment”), we created a database with all individuals in the Swedish population born in Sweden 1955-1990. We also included the number of years, during ages 0-15, that individuals resided in the same household as their biological father. We thereby were able to define two kinds of families i) “not-lived-with” father families (offspring never resided for more than 1 year in the same household or in the same community as their biological father); ii) “lived-with” father (offspring resided a minimum of 13 year in the same household as their biological father. We performed a linear regression model with the Z-score for education trait in offspring as outcome and the Z-score for education in father, type of father, and their interaction as predictors. We used the interaction term as the difference of effect between genes only and genes + environment. The same approach was performed for half-siblings where we compared those who were reared together versus reared apart. (For parents this component was 0.87 and for siblings it was 0.76)  For each relative we then calculated the product using the three components: mean Z-score, cohabitation effects, proportion of shared genetic effects (0.003125 -0.5) with the proband. Then we average this product across all relatives to a proband. Then we corrected for the number of relatives. We multiplied the results from the previous step with a shrinkage factor. (Shrinkage factor (SF): B/(B+A/C). It produces more shrinkage if B and C are small and A is large. (A) = the variance of the z-score of the disorder across all relatives, (B) = the variance in the mean z-score across all probands,(C) = the weighted number of relatives for each proband. We standardized the risk score by year of birth and county of the proband into a z-score with mean 0 and SD 1. This was then used as the GAEA in the analyses. |
| Social welfare recipient | Longitudinal integrated database for health insurance and labour market studies (LISA) | Social assistance is defined as financial support under the Social Services Act. You can receive support for your upkeep and for other items that you need to have a reasonable standard of living. Examples of common situations when social assistance is given: As an income supplement to low-income families; for unemployed when other unemployment assistance is not provided or is insufficient; when sickness benefits are insufficient or not provided; to those who are bound by the children in the home and cannot get childcare and therefore not can seek work. The variable is recorded at the family level, which means that all individuals in a family with social assistance will, in this report, be counted as recipients of Social welfare . |
| Deprivation | Longitudinal integrated database for health insurance and labour market studies (LISA), Register of Total Population | For every year an individuals is registered at a specific DESO area. The DeSO areas divides Sweden into 5,983 areas and have between 700 and 2,700 inhabitants. The division takes into account the geographical conditions so that the boundaries follow, for example, streets, waterways and railways. Important building blocks used to create DeSO are urban areas and electoral districts. For each of the DeSO area we created a neighborhood social deprivation (SD) index based on register data for all residents in the neighborhood aged 25-64. We used deprivation indicators used by past studies to characterize neighborhood environments and then used a principal component analysis to calculate a z-score. The following four variables were included: low educational status (defined as less than 10 years of formal education); low income (from all sources, including that from interest and dividends, which was defined as less than 50% of individual median income); unemployment (defined as not employed; excluding full-time students, those completing compulsory military service, and early retirees); and social welfare assistance. We define high deprivation as above one StD from the mean value. |
| Early Retirement | Longitudinal integrated database for health insurance and labour market studies (LISA) | Early retirement is a combination of several different variables from the Swedish registers. Until year 2002, Early Retirement Pension was paid to people aged 16- 64 and granted when their working capability was deemed to be permanently reduced by at least one quarter due to medical reasons. The early retirement variable is also composed of temporary disability pensions paid to individuals whose working capability was not expected to be permanent but was expected to persist for a considerable time. From 2003, the rules for Early Retirement Pension and Temporary disability pension changed and these types of compensations were changed into sickness compensation (for individuals 30-64) and activity compensation (for individuals 19-29). The qualification rules were similar but activity compensation was supposed to be limited in time. |
| Sickleave | Longitudinal integrated database for health insurance and labour market studies (LISA) | Several of the sickness allowances (but also the parental allowance) are based on a “sickness benefit-based income” (Swedish: “sjukpenninggrundande inkomst-SGI”), which is currently 97% of a person’s individual annual earnings. Benefits typically correspond to 80% of this amount (0.80 * 0.97) but are capped at a lower level for high-income earners (capped at 7.5 basic amounts annually for sick leave compensation). The basic amount (Swedish: “basbelopp”) has changed over the years, increasing about 50%  During the first 14 days of sick leave, the employers are responsible for sick pay. Because this pay cannot be differentiated from ordinary salary, Statistics Sweden cannot identify sick leave ≤ 14 days. If a sick leave episode is ≤ 14 days, the episode does not enter into the sick leave statistics in LISA. Previously, the first qualifying day had no cash benefits (Swedish: “karensdag”). On Jan 1, 2019 the government removed the qualifying day as some employees were affected more than others (especially people working evenings and weekends), and instead introduced a qualifying deduction. After the first 14 days, the individual can apply for sick leave benefit (Swedish: “sjukpenning”; SjukPA and SjukPP). For repeated sick leave episodes with a short interval in between and for patients with a chronic disease, the responsibility of the employer to compensate the first 14 days can be waived. The self-employed can choose to have 2 qualifying days or more without cash benefits (self-employed who are sick ARE paid by the Swedish Social Insurance Agency). |

**Table 3a Results from the Factor Analysis:** Polychoric correlation between the included indicators

|  | Social Welfare | Deprived area | Sick Leave | Early Retirement |
| --- | --- | --- | --- | --- |
| Social Welfare | 1 | 0.45 | 0.18 | 0.42 |
| Deprived area |  | 1 | 0.09 | 0.28 |
| Sick Leave |  |  | 1 | 0.12 |
| Early Retirement |  |  |  | 1 |

**Table 3b Results from the Factor Analysis**: Factor loadings for each indicator

|  | Factor loading |
| --- | --- |
| Social Welfare | 0.672 |
| Deprived area | 0.182 |
| Sick Leave | 0.051 |
| Early Retirement | 0.171 |

**Table 4 Calculation of the Familial Genetic Risk Score (FGRS)**

|  |
| --- |
| The dataset for the calculations includes:  Column1 = Identification number of the proband (Born 1932-1995)  Column2 = Identification number of the relative (1st to 5th degree relatives)  Column3 = Proportion of shared additive genetic effects (0.03125 to 0.50) with the proband  Column4 = Year of Birth of relative  Column5 = Sex of relative  Column6 = Age at registration for trait  Column7 = Age at end of follow-up (2018-12-31 or age at death, or age at emigration whichever came first) |
| Step 1: Using all unique relatives with a registration for the disorder, we non-parametrically estimated the distribution of Age at first registration. The empirical distribution is used to obtain weights for relatives without a registration for the disorder, in order to account for the proportion of the time-at-risk period they had completed at the end of follow-up. For example, for relatives at age x at end of follow-up, the weight corresponds to the proportion of relatives registered for the trait that had been registration at age x. For relatives born prior to 1958 we subtracted age at the end of follow-up with the following formula: 1958 - Year of birth of relative. This modification was done in order to control for registration effects (i.e, most registers in Sweden start in 1973 suggesting that relatives from early birth cohorts do not have the possibility to be registered at younger ages). Note that all relatives with the disorder are weighted one. |
| Step 2: Transform the binary variable (trait yes/no) into a z-score based on the threshold for each trait. The underlying liability of the individual is not assessable. Instead we estimated the mean of the underlying liability to obtain sex and birth decade specific Z-scores for relatives with the trait registration and relatives without the trait. We generate n random numbers from a N(0, 1) distribution and estimate the mean for relatives registered with the disorder (i.e., mean of the observations above the threshold) and for relatives without a registration (i.e., mean of all observation below the threshold). The thresholds are calculated for each decade of birth and sex. |
| Step 3: Correct for cohabitation effects. To estimate the cohabitation effect (i.e. “shared environment”), we created a database with all individuals in the Swedish population born in Sweden 1955-1990. We also included the number of years, during ages 0-15, that individuals resided in the same household as their biological father. We thereby were able to define two kinds of families i) “not-lived-with” father families (offspring never resided for more than 1 year in the same household or in the same community as their biological father); ii) “lived-with” father (offspring resided a minimum of 13 year in the same household as their biological father. We performed a logistic regression model with the binary trait in offspring as outcome and the binary trait in father, type of father, and their interaction as predictors. We used the interaction term as the difference of effect between genes only and genes + environment. The same approach was performed for half-siblings where we compared those who were reared together versus reared apart. The following interaction terms were used in the calculations for each of our disorders:   \|  \| Parent/Children \| Siblings \| \| --- \| --- \| --- \| \| MD \| 0.80 \| 0.85 \| \| AD \| 0.87 \| 0.81 \| \| BD \| 0.67 \| 0.77 \| \| SZ \| 0.93 \| 0.84 \| \| AUD \| 0.99 \| 0.69 \| \| DUD \| 0.92 \| 0.52 \| \| ADHD \| 0.42 \| 0.81 \| \| ASD \| 0.83 \| 0.61 \| \|  \| \| \| |
| Step 4: Calculate the product for each relative using the four components:   1. Z-score (reflecting sex and year of birth adjusted rates) 2. Weight (reflecting the proportion of risk period they had completed) 3. Cohabitation effects 4. Proportion of shared genetic effects (0.03125 – 0.5) with the proband |
| Step 5: Average the product calculated in step 4 across all relatives to a proband |
| Step 6: Correct for the number of relatives. We multiplied the results from step 5 with a shrinkage factor. Shrinkage factor (SF): B/(B+A/C). It produces more shrinkage if B and C are small and A is large.   1. the variance of the z-score of the disorder across all relatives, 2. the variance in the mean z-score across all probands, 3. the weighted number of relatives for each proband (sum of Column 3 across each proband). |
| Step 7: Correct for difference by year of birth and county differences. There are 21 counties in Sweden. For each proband we used the county they had resided in during the maximum number of years (measured from 1969 and onwards) We standardized the risk score by year of birth and county of the proband into a z-score with mean 0 and SD 1. |

**Figures 2 – Social Outcome of AD Cases With and Without Comorbid Cases of MD**


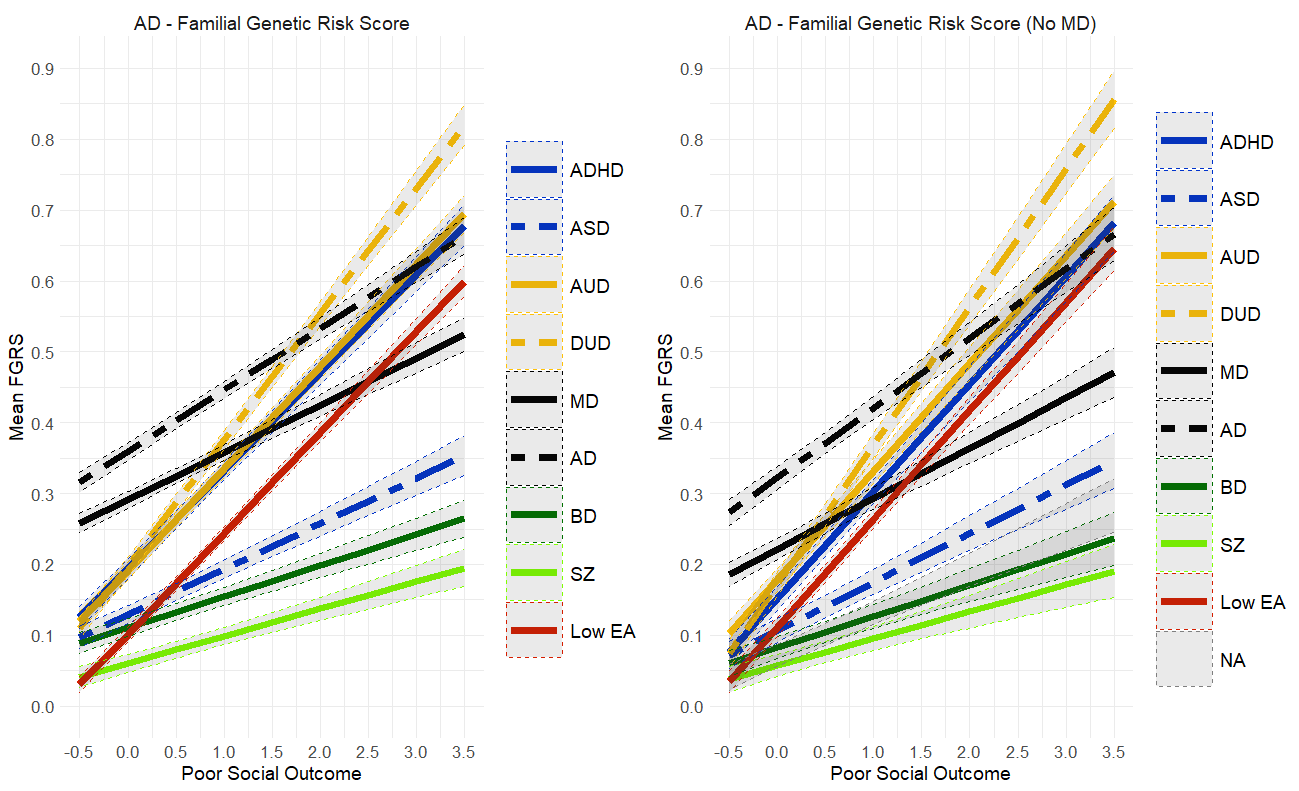


**Figure 3 – Social Outcome of MD Cases With and Without Comorbid Cases of ADS**


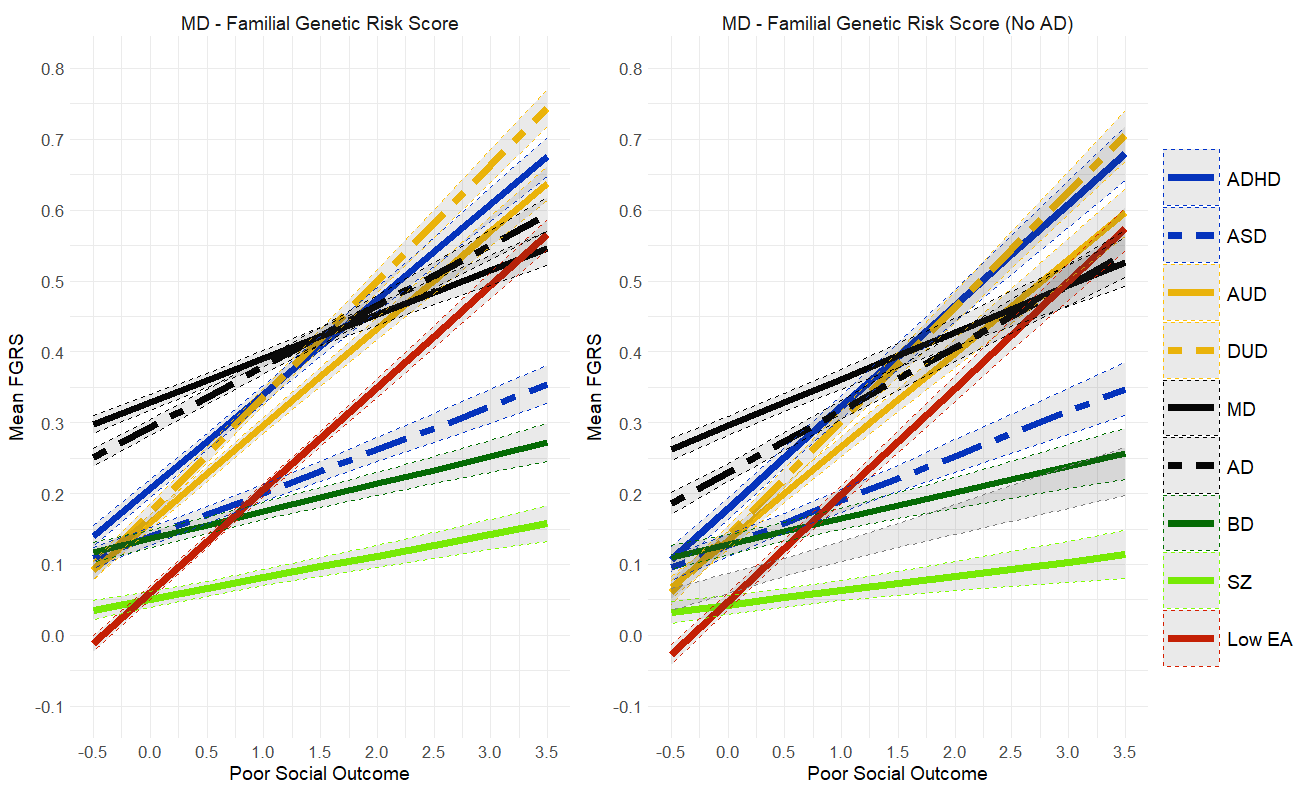


**Figure 4 – Social Outcome of BD Cases With and Without Comorbid Cases of SZ**


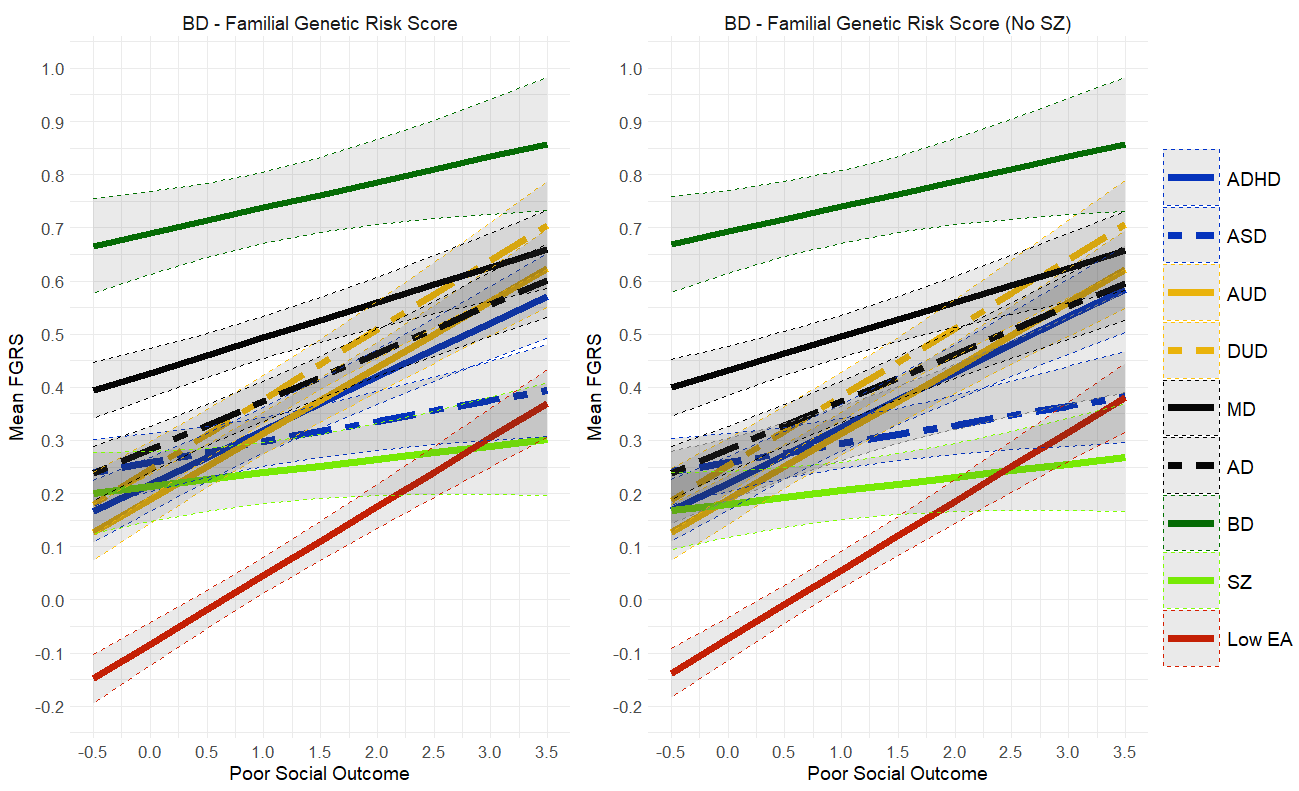


**Figure 5 – Social Outcome of SZ Cases With and Without Comorbid Cases of BD**


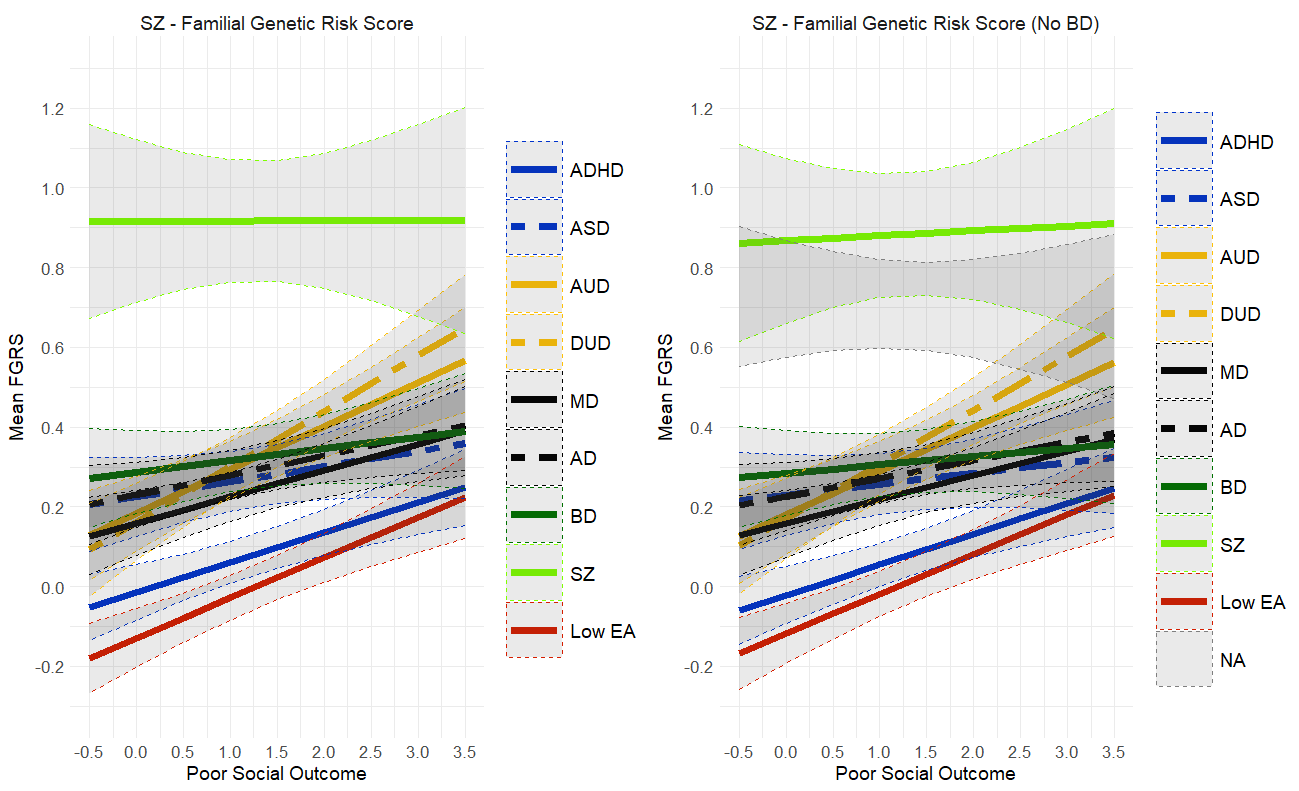


**Figure 6 – Social Outcome of AUD Cases With and Without Comorbid Cases of DUD**

**
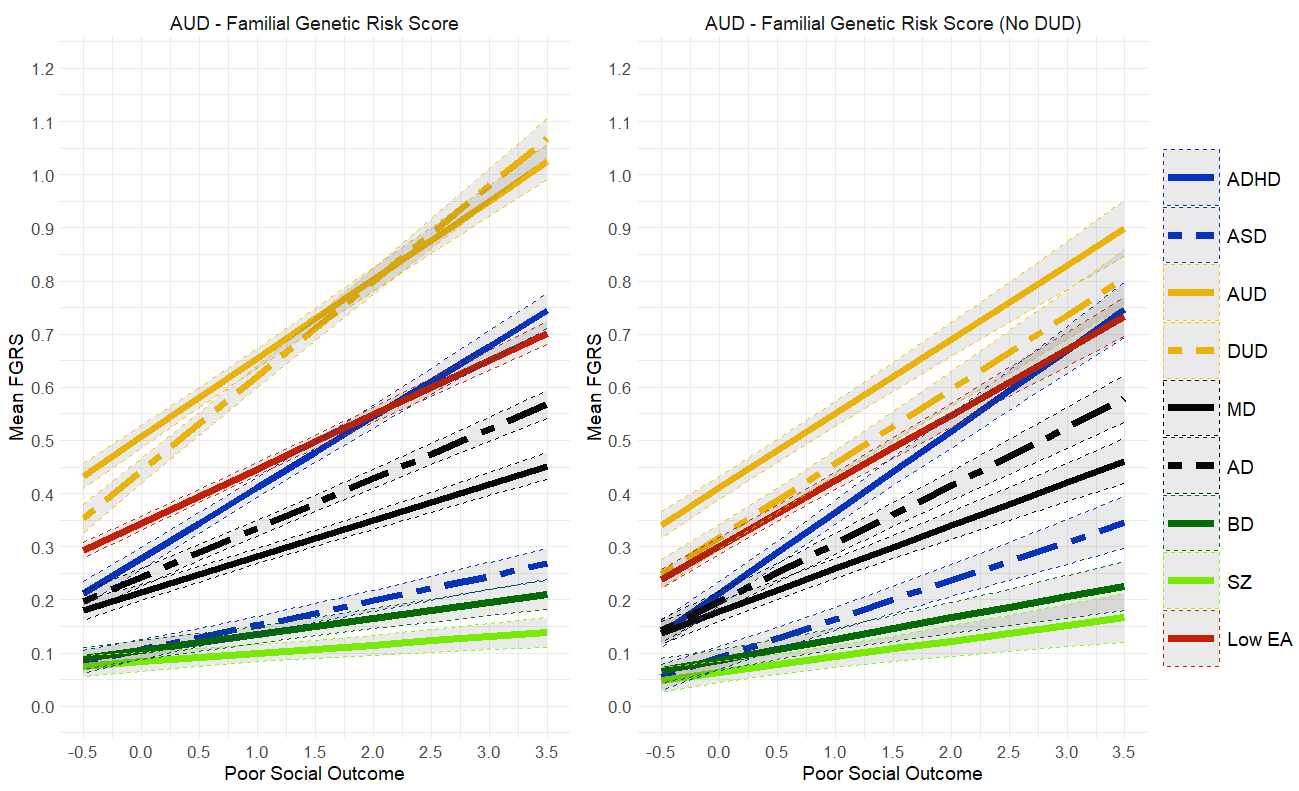
**

**Figure 7 – Social Outcome of DUD Cases With and Without Comorbid Cases of AUD**


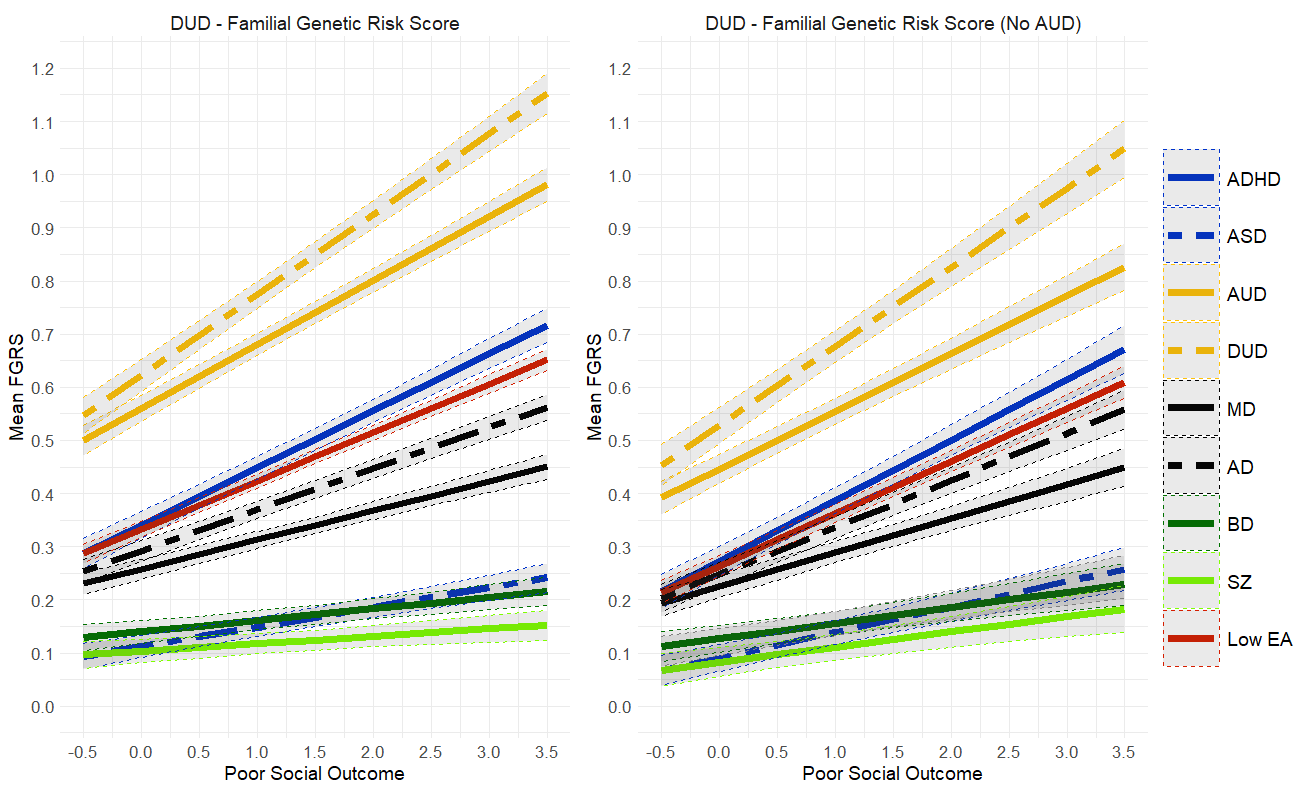


**Figure 8 – Psychiatric Outcome of BD Cases Without Comorbid Cases of SZ and SZ Cases Without Comorbid Cases of BD**


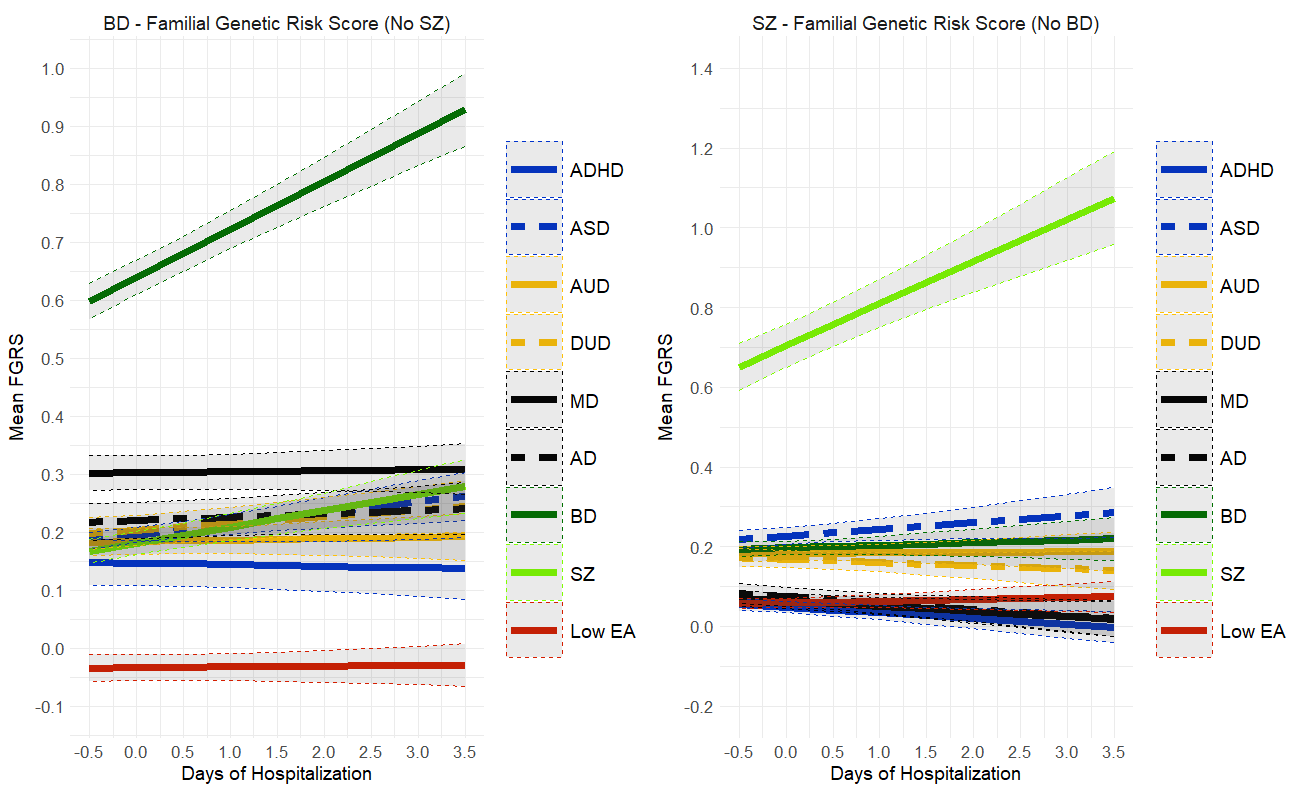


**Figure 9 – Social Outcome of Male BD Cases and Female BD Cases**


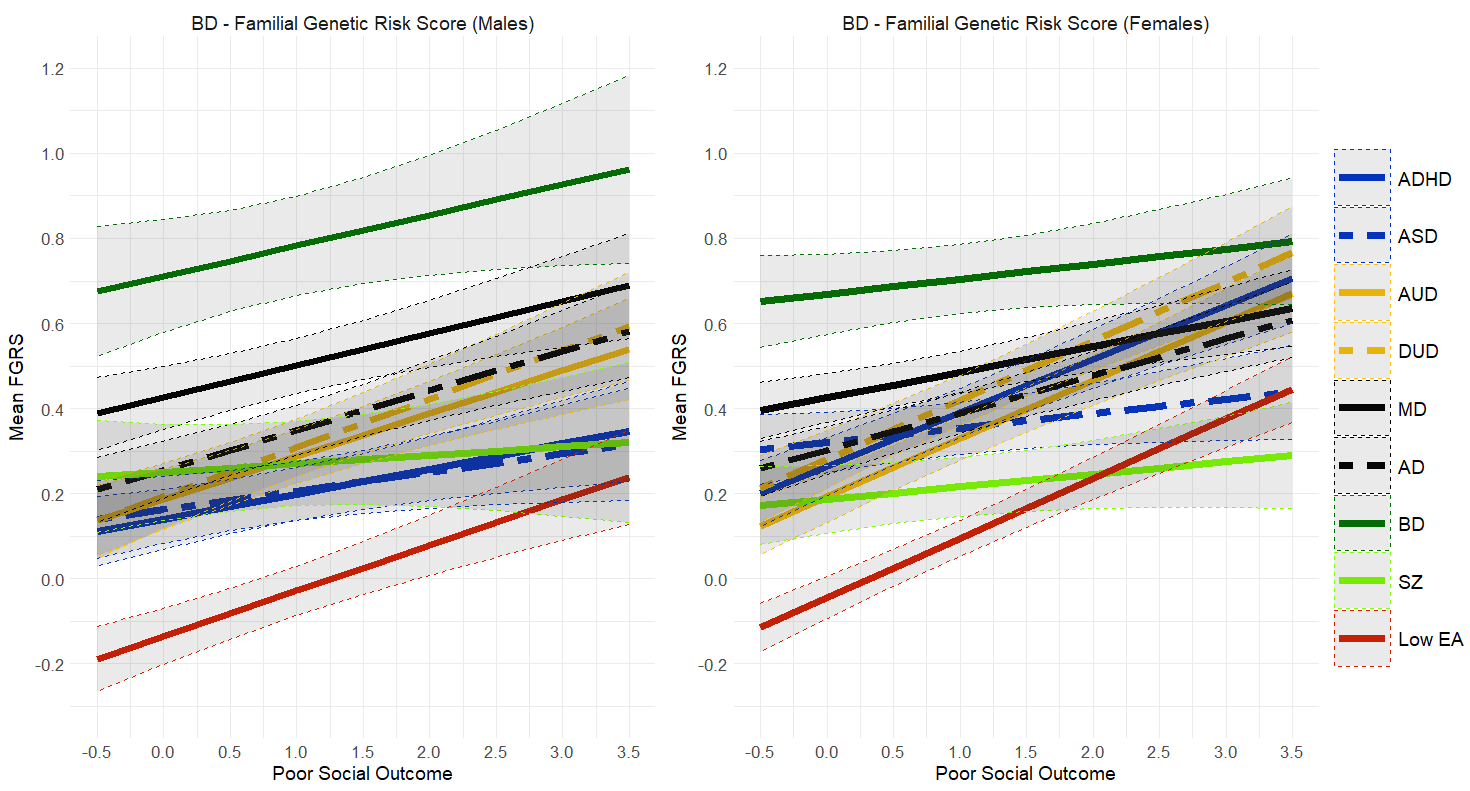


**Figure 10 – Social Outcome of Male SZ Cases and Female SZ Cases**


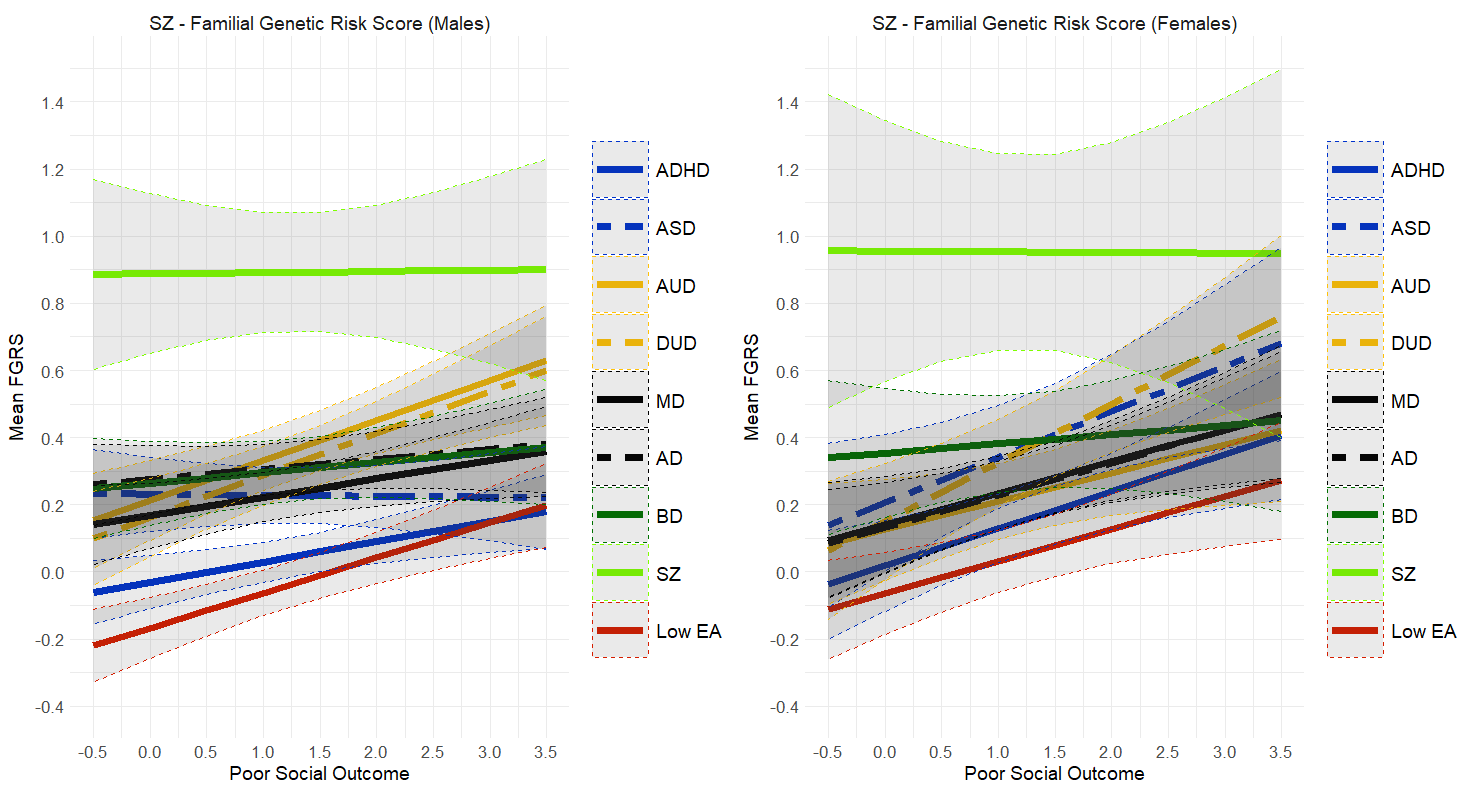


**Figure 11 – Social Outcome of Male AUD Cases and Female AUD Cases**


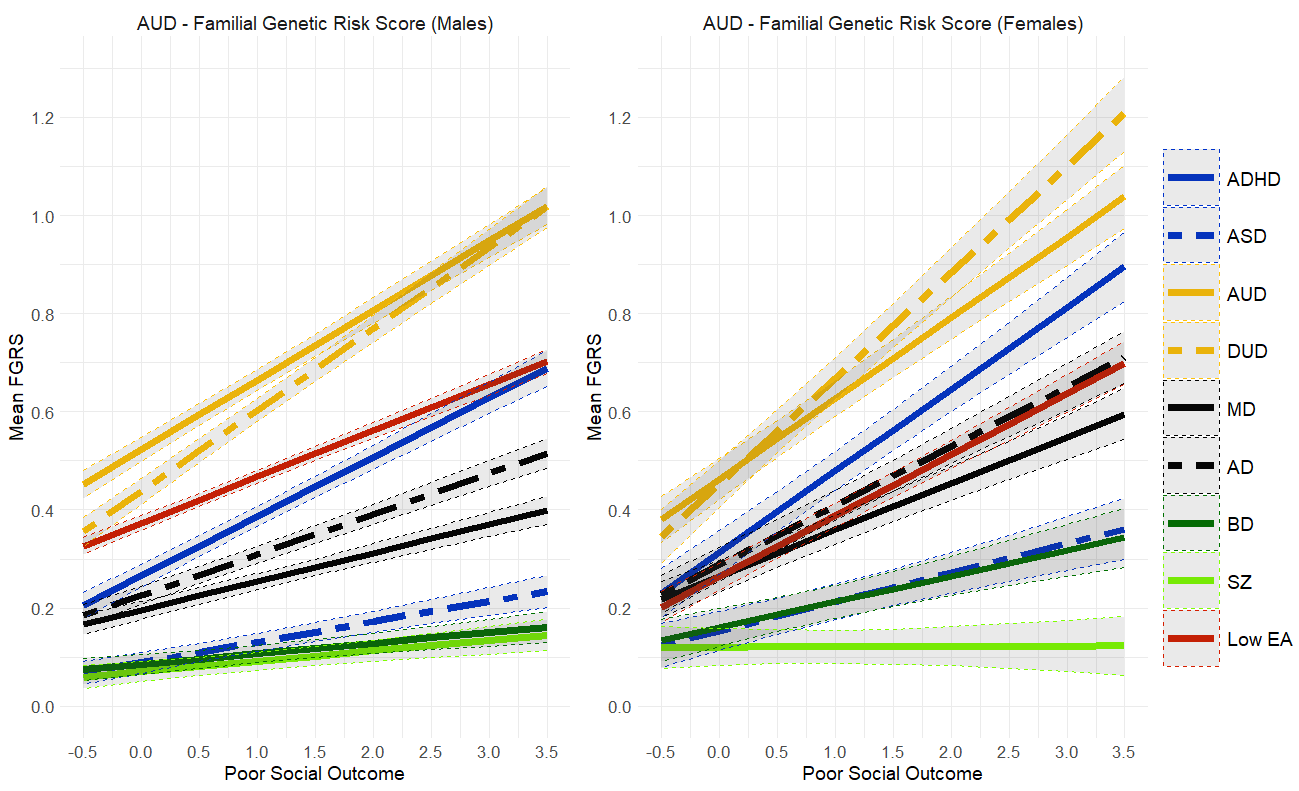


**Figure 12 – Social Outcome of Male DUD Cases and Female DUD Cases**


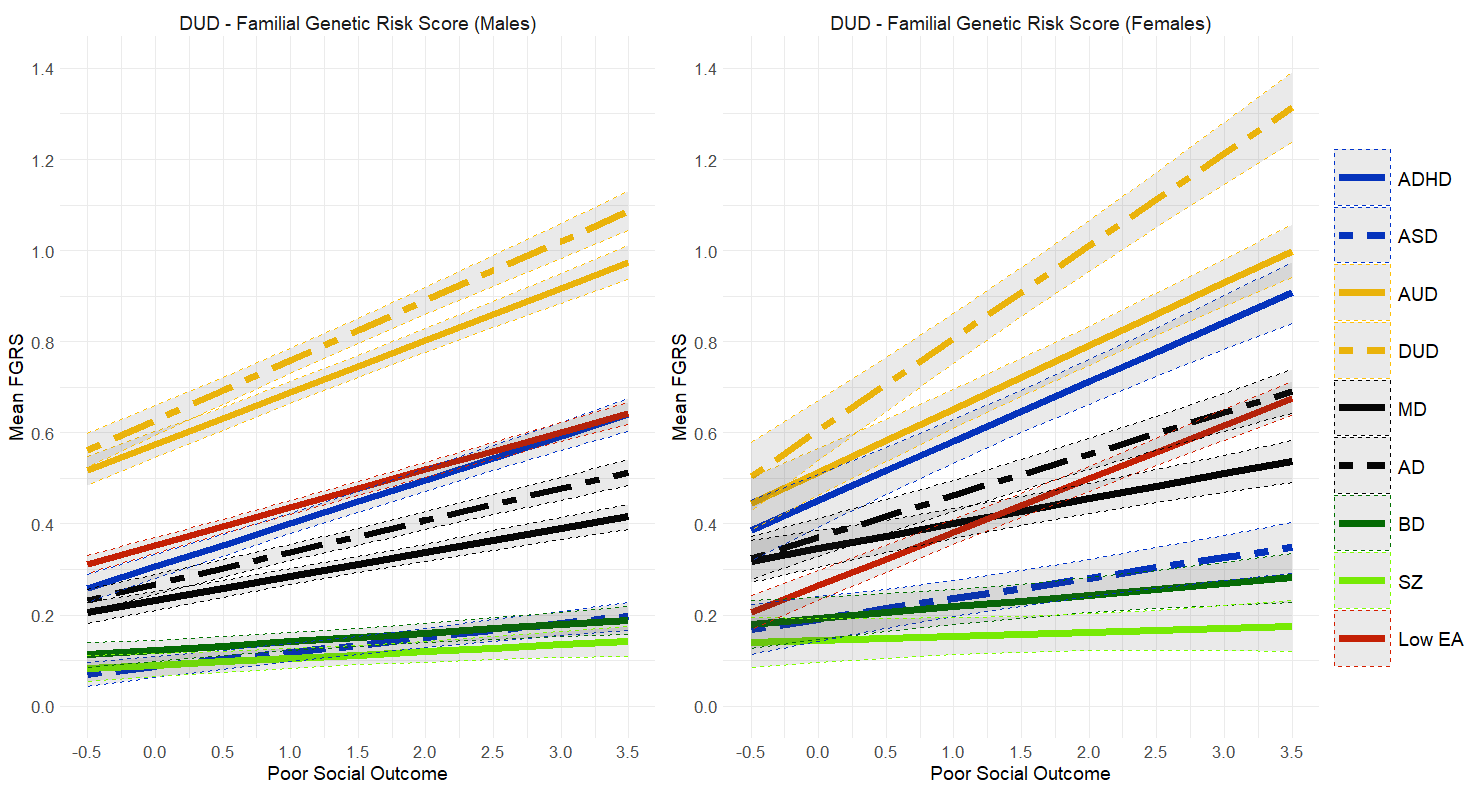


**Figure 13 – Psychiatric Outcome of Male BD Cases and Female BD Cases**


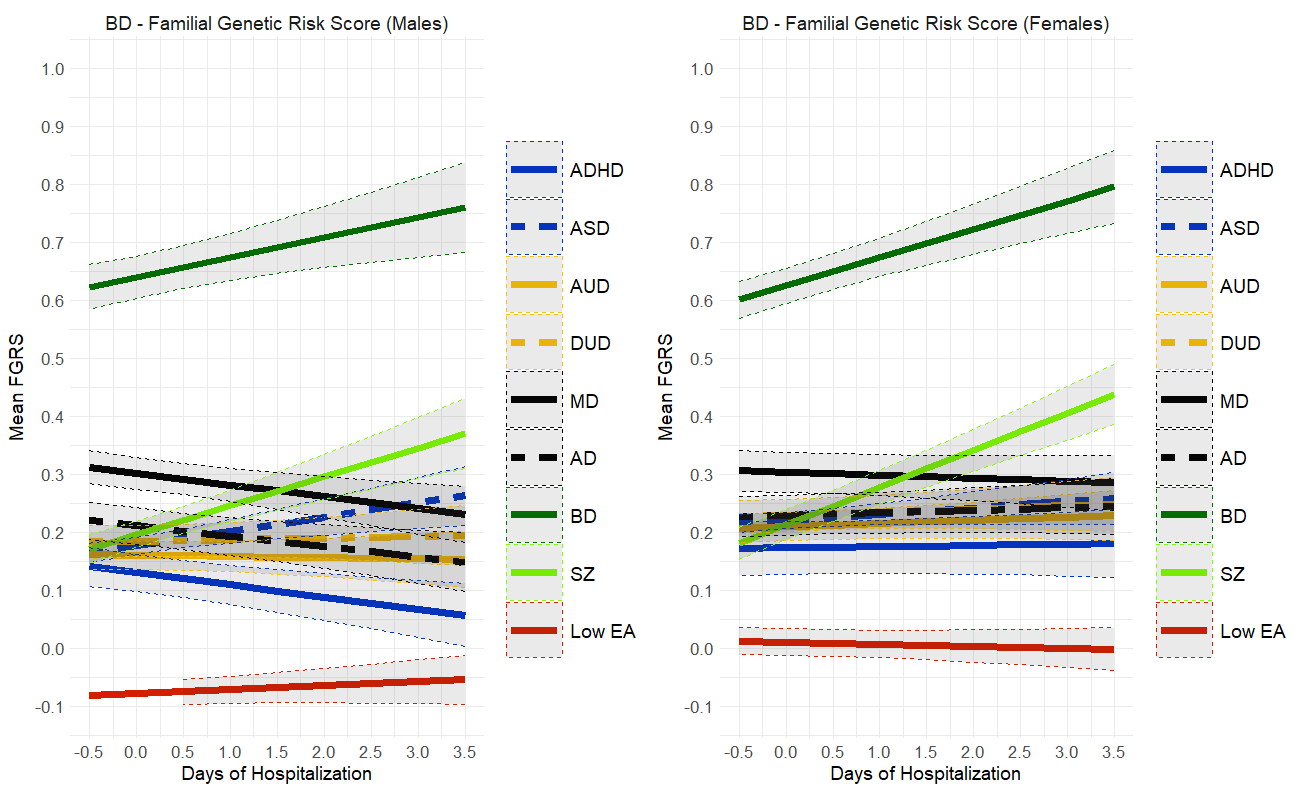


**Figure 14 – Psychiatric Outcome of Male SZ Cases and Female SZ Cases**


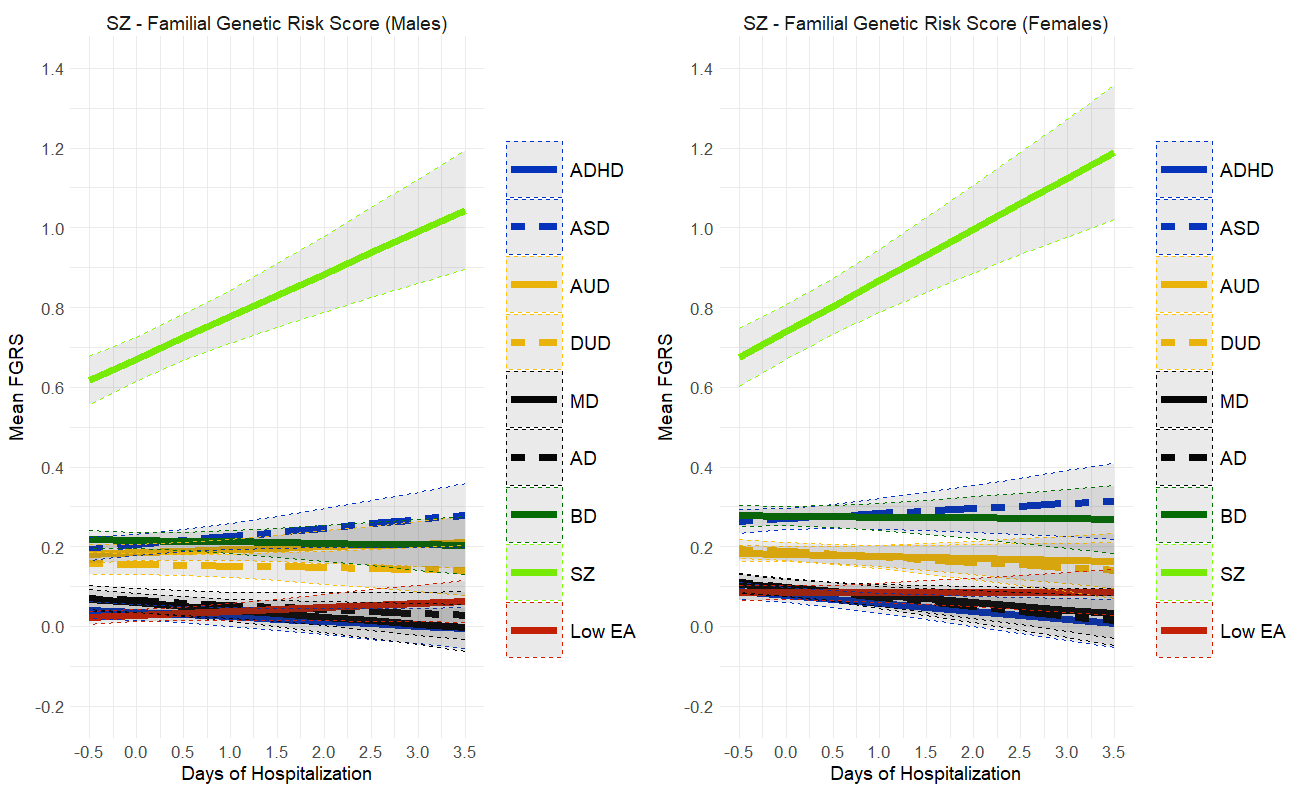

Supplement: Kendler et al. supplementary material [file S0033291725101116sup001.docx]
